# Impact of Covid-19 lockdowns on the anthropometric development in primary school children in the Rhein-Neckar Region, Germany

**DOI:** 10.1186/s40795-024-00886-2

**Published:** 2024-05-29

**Authors:** Azat Samigullin, Gabriel Groß, Jana Gählert, Sandra Buchenberger, Michael Morcos, Rainer Schwertz, Rickard Öste, Erhard Siegel, Per Humpert

**Affiliations:** 1 Starscience GmbH, Heidelberg, Germany; 2 Stoffwechselzentrum Rhein-Pfalz, Mannheim, Germany; 3 Medical Faculty Mannheim, University of Heidelberg, Mannheim, Germany; 4 Medical Faculty Heidelberg, University of Heidelberg, Heidelberg, Germany; 5 Gesundheitsamt Rhein-Neckar-Kreis, Heidelberg, Germany; 6 Aventure AB, Lund, Sweden; 7 St. Josefskrankenhaus, Heidelberg, Germany; 8 Adipositasnetzwerk Rhein-Neckar e. V., Heidelberg, Germany

**Keywords:** Schools, Community, Child health, Pediatrics, Child development, Obesity, Nutrition, COVID-19, Lockdown

## Abstract

**Background:**

Published data suggests that lockdowns during the COVID-19 pandemic may have negatively affected children’s weight development. This study aims to assess the prevalence of overweight and obesity after the COVID-19 lockdowns as well as anthropometric development among primary school children in the Rhein-Neckar Region, Germany.

**Methods:**

In this cross-sectional study, schools were selected in cooperation with the local health authority to include different socioeconomic backgrounds. Participation was voluntary at school and individual level, requiring written informed consent from legal guardians. Study visits in schools were conducted between October 2021 and July 2022. Anthropometric data from nationally recommended medical examinations at 4 years (U8) and 5 years (U9), data on nutrition, physical activity, and socioeconomic data was collected using questionnaires. zBMI and weight category were calculated based on German reference data.

**Results:**

256 children with a mean age of 8.0 years (7.1–9.3 years) were included in the study. Most participants were from households with an above average income. 5.1% of the children were overweight, 4.7% were obese, 15.6% were underweight, and 74.6% were normal weight with a mean zBMI of -0.25 (SD 1.10), which is significantly lower than the mean zBMI of the German reference population (*p* < 0.001). No significant changes in zBMI were observed between U8 and U9 (*p* = 0.16). The mean zBMI decreased by 0.17 (SD 0.72) between U9 and the study visit (*p* = 0.02). A zBMI decrease of 0.5 was documented for the subgroup of overweight and obese children (*p* = 0.028) as well as a decrease of 0.23 (SD 0.63) for the normal-weight children subgroup (*p* < 0.001).

**Conclusions:**

Contrary to previous reports mean zBMI decreased significantly in the children studied. No significant changes in zBMI were observed between U8 and U9 examinations, which supports the hypothesis that the decrease in zBMI could be attributed to lockdown measures. The study was registered at clinicaltrials.gov on September 21st 2021 under the registration number NCT05077059.

**Supplementary Information:**

The online version contains supplementary material available at 10.1186/s40795-024-00886-2.

## Introduction

In recent decades the prevalence of obesity and overweight of children has increased throughout the world [1, 2]. Children with overweight and obesity suffer a variety of health issues related to their weight and have a high risk of remaining overweight or obese in adulthood [1, 2]. In Germany, according to the KiGGS study (“Studie zur Gesundheit von Kindern und Jugendlichen in Deutschland” / “German Health Interview and Examination Survey for Children and Adolescents”, conducted 2014-17), 15.4% of children are overweight and 5.9% are obese [3]. In 7–10 year old girls 14.9% are overweight; in 7–10 year old boys 16.1% are overweight [3]. These prevalences include obesity, which is 4.7% for girls and 6.8% for boys [3]. As in adults, body weight and composition are strongly affected by diet and levels of physical activity [1, 4]. Government COVID-19 measures in Germany interfered with approximately two thirds of the 270 regular school days between January 2020 and May 2021 (beginning of pandemic to end of measures), with school closings beginning in March and schools remaining completely closed for almost one third of this time [5]. Depending on individual school policy, recommendations from local health authorities national recommendations and guidelines valid at the different timepoints during the pandemic, children in primary schools had to wear masks, were limited in physical activity due to cancellation of sports lessons, were not allowed to mix with other classes and missed many lessons either due to testing positive for COVID-19 or an increased sick leave of teachers [5]. Initial scientific reports suggest that the COVID-19 pandemic and measures to contain its spread lead to a significant learning loss [6, 7] as well as negative psychological impact [8, 9] – studies from Germany show that around two thirds of children reported a deterioration of quality of life or felt burdened by the measures [10–14].

It has been suggested early in the pandemic that these measures may result in an unhealthy weight gain in school children [15]. Published studies to date support a general disproportional weight gain during the pandemic [16], according to studies conducted in Germany, children appeared to disproportionally gain weight during the pandemic measures [17]with obese children being more profoundly affected [18, 19]. Weight gain in children was also reported in the US [20, 21], Japan [22], Chile [23], and in preschoolers in Sweden [24], while showing mixed results in Israel [25–27], where weight development depended on age and socioeconomic status. A meta-analysis, showed a very-low-certainty weight gain with a high heterogeneity [28]. Eating habits, increased screen time and reduced physical activity were identified as factors connected to disproportional weight gain [19, 29]. The definitions of overweight and obesity in children are centered around the standardized Body-Mass-Index (BMI) but additional related variables such as waist and hip circumferences are gaining importance [30].

This study aims to assess the prevalence of overweight and obesity, physical activity and eating habits as well as the retrospective anthropometric development in the context of COVID-19 containment measures among a sample of primary school children (second grade) in the Rhein-Neckar Region, Germany.

## Materials and methods

Schools from different socioeconomic backgrounds from both urban and rural regions were selected in the Rhein-Neckar region in cooperation with the local health authorities. If head teachers agreed to participate, an appointment for a school visit was set, and suitable rooms inside the schools were allocated. Participation in the study was voluntary at school and individual level. Written consent from parents or legal guardians was mandatory for participation in the study and was available in five languages (German, English, Turkish, Russian, and Arabic). Incentivization or compensation of any sort for participation was not permitted by the school authority. Data collection was pseudonymized. All examinations were performed in compliance with COVID-19 measures mandated at the time. The study was designed to detect an obesity prevalence of 4% with the confidence level of 95% and a precision of 2% requiring 369 participants [31].

School visits were conducted between October 2021 and July 2022 with an interruption due to lockdown restrictions and high COVID-19 incidences. A total of 256 children of 1,401 invited to participate took part in the study. Recruitment had to be paused from January to April due to an increased number of cases and schools not having the resources to take part in the study at that timepoint; 135 children were recruited before and 121 after the interruption.

Weight was measured using a calibrated SECA 877 or a calibrated SECA 899 scale (precision of 0.1 kg), height was measured using the SECA 437 adapter and a SECA 217 stadiometer in combination with the scales (precision of 0.1 cm). Circumferences were measured using new SECA 201 measuring tapes (precision of 0.1 cm) acquired specifically for this study. The devices were setup and measurements were performed according to the manufacturer’s recommendations. The measurements included size in centimeters, weight in kilograms, waist circumference, hip circumference, and arm circumference of the dominant arm. Weighing was performed after shoes and outdoor clothes were removed. Indoor clothes were still worn. The weight of clothing, still worn by child was estimated by the researchers using a table with the weights of clothing typically worn by children and was later subtracted from the weight on the scale (created beforehand by weighing different kinds of children’s clothing). When children were wearing thick or very thick clothes, researchers were instructed to subtract 0.5 cm or 1.0 cm from the measurement at their discretion in order to correct for the thickness of the clothing worn, these modalities were clearly communicated to the study team and practiced before the first visit to be as consistent as possible. Data from the nationally recommended pediatric U8 and U9 examinations, which are conducted at approximately age 4 and 5 years in Germany were collected together with the anthropometric data of the parents using a questionnaire as well as the parent’s subjective assessment of the childs weight status and weight status development during the lockdowns. Data was entered into the database with a precision of one decimal for weight, height and BMI.

Physical activity was assessed using an adapted PAQ-C questionnaire, which was completed by a study group member together with the child and a physical activity questionnaire completed by parents, which included the question regarding number of days with over 60 min of physical activity, which is a WHO criterium [32]. Socioeconomic status was assessed using a questionnaire and included household income, parental education and migration background. If only one parent was filling out the questionnaire, it was assumed that this information in regards to the other parent was known by the parent filling out the questionnaire. The questionnaire can be found in Annex 1.

Nutrition was assessed using a short self-designed food questionnaire which asked whether children adhere to a vegetarian or vegan diet, how often parents cook in the household, how often the family eats out at restaurants or orders food, if parents smoke and what beverages the child consumes. The 3-day food diary was completed day by day by hand in the preferred language of the family. Data from the food diary was entered intoPRODI® Software (version 6.10 compact plus, Nutri-Science GmbH), which uses the German List of foods (Bundeslebensmittelschlüssel version 3.02) to calculate calories, macronutrients and micronutrients in the foods recorded in the food diary. Consumption of medication, vitamins or supplements was not collected.

A total socioeconomic status score was calculated similar to procedure in the KIGGS-study [33]. Highest education level of the father and mother each being assigned a score between 1 and 6 as well as the household income ranging from 1 to 5, resulting in a maximum total score of 17. Based on this calculated value, the population was divided into five quintiles. The lowest quintile was designated as “lower SES,” the highest as “higher SES,” and the remaining three quintiles were referred to as “mid-range SES.”

IBM SPSS Statistics, version 29, was used for all statistical analyses. P-values < 0.05 were considered statistically significant. Sample sizes are reported alongside results, when data is missing. BMI was calculated as weight in kilograms divided by their height in meters squared (weight (kg) / [height (m)]^2^) and used to derive age-adjusted z-values and percentiles for weight, height and BMI. Age and sex adjusted zBMI values were calculated using the equation zBMI = ((BMI/M)^L-1)/(L*S), whereby the values for L, M and S were derived from reference tables. Reference tables used were percentiles derived from the KiGGS study [34]. Percentile cut-offs were also derived using the same reference tables. According to German national definitions, overweight is defined as BMI above the 90th percentile; obesity as BMI above the 97th percentile and underweight as below the 10th percentile [35]. Waist to hip ratio was calculated by dividing the circumference of the waist by the circumference of the hips. Normalized data for this age group in Germany is available for BMI but not for waist, hip and arm circumferences or waist-to-hip ratio. zBMI development from U8 to U9 examination to this study was assessed using a paired t-test. For normally distributed variables Pearson correlations were calculated, for non-normally distributed variables Spearman correlations were calculated. An explorative stepwise regression analysis was performed using variables with significant correlations to zBMI change between U9 examination and the timepoint of study. The stepwise regression included zBMI from the U9 examination (since it is the baseline) and all variables, for which significant correlations were found. Sensitivity analyses were performed for central variables using an independent t-test.

## Results

Anthropometric data was obtained for all 256 participants (100%). The Physical Activity Questionnaire for Children (PAQ-C) was administered to 253 participants (99%). Parent-reported physical activity data and socioeconomic status (SES) information were available for 179 participants (70% of the sample). Furthermore, 172 participants (67%) completed a short nutritional questionnaire, providing valuable insights into their dietary habits. Lastly, a 3-day food record was collected from 142 individuals (55%).

Of the 256 participants 122 were boys (48%) and 134 were girls (52%). The median age was 8.0 years (7.1–9.3 years) and the interquartile range (IQR) was 7.7–8.3 years. The median Body Mass Index (BMI) was 15.6 and the median z-score BMI (zBMI) -0.33 with an IQR of -0.93 to 0.40 (*n* = 256). The mean zBMI for the entire cohort was − 0.25, with a standard deviation of ± 1.1 zBMI (*n* = 256). Data on height, weight, hip, waist and arm circumferences, waist to hip ratios as well as the corresponding z-values (where German reference values are available) and weight class distribution are presented in Table 1.

**Table 1:** Summary Statistics of Anthropometric Measurements for all participants as well as designations according to current German growth charts. Data is presented as mean (SD) unless stated otherwise. Underweight according to German growth standards is defined as < 10th percentile; Normal weight ≥ 10th and < 90th percentile, overweight ≥ 90th and < 97th percentile and obesity as > 97th percentile. The German growth charts do not include waist, arm or hip circumferences or waist-to-hip ratio, thus z-values are not available for these

	All participants	*N*	Girls	*N*	Boys	*N*
Height (cm)	131.7 (6.5)	256	130.8 (6.5)	136	132.7 (6.3)	120
Weight (kg)	28.6 (7.5)	256	28.3 (7.9)	136	28.9 (7.1)	120
BMI	16.3 (2.9)	256	16.3 (2.9)	136	16.3 (3.0)	120
U9 BMI	15.4 (1.4)	170	15.4 (1.4)	98	15.3 (1.4)	72
U8 BMI	15.6 (1.3)	170	15.5 (1.2)	96	15.8 (1.4)	74
zBMI	-0.25 (1.10)	256	-0.16 (1.06)	136	-0.34 (1.15)	120
Underweight (< 10P)	40 (16%)	256	19 (14%)	136	21 (18%)	120
Normal weight (≥ 10P; ≤90P)	191 (75%)	256	105 (77%)	136	86 (72%)	120
Overweight (> 90P; ≤97P)	13 (5%)	256	9 (7%)	136	4 (3%)	120
Obese (> 97P)	12 (5%)	256	3 (2%)	136	9 (8%)	120
zBMI Subgroup	-0.35 (0.99)	168	-0.23 (0.99)	97	-0.51 (0.98)	71
U9 zBMI	-0.18 (1.0)	168	-0.13 (0.97)	97	-0.25 (1.05)	71
U8 zBMI	-0.10 (0.99)	170	-0.16 (1.05)	96	-0.03 (1.04)	74
Waist circumference (cm)	58.9 (58.9)	256	58.2 (7.3)	136	59.7 (7.9)	120
Hip circumference (cm)	62.8 (62.8)	256	62.6 (7.7)	136	63 (7.9)	120
Arm circumference (cm)	19.8 (19.8)	256	19.9 (2.2)	136	19.8 (2.4)	120
Waist to Hip Ratio	0.94 (0.04)	256	0.93 (0.04)	136	0.95 (0.03)	120

Since U8 and U9 examinations were only available for a subgroup, weight change could only be assessed in 168 children. The prevalence of obesity in this group was 1.7%, of overweight 4.0%, 75.7% were normal weight and 18.5% underweight, thus lower than for the total group. The difference between the zBMI of the subgroup, where U-examination data was available (mean − 0.35; *n* = 168) and where it was not (mean − 0.05, *n* = 88) was not statistically significant (*p* = 0.06). zBMI did not change significantly from U8 examination to U9 examination (-0.10 ± 0.99 vs. -0.18 ± 1.00). A significant zBMI decrease was observed from U9 to the present study (-0.35 ± 1.00) with a delta of 0.17 (*p* = 0.02), and from U8 examination to the present with a delta of 0.25 (*p* < 0.01). The zBMI reduction was mainly reflected by an increase from 12.7 to 18.5% in the proportion of children classified as underweight.

86% of the children did not adhere to any special diet. In 35% of the households fresh food was cooked every day, in 29% of households 6 days a week, 15% five days a week, 11% 4 days a week, 4% 3 days a week, 5% on two days a week and 1% never. In median restaurant food was consumed once a week (IQR 0–1).

Based on the data calculated from food diaries, the majority of children did not reach the recommendations of the German Society for Nutrition (DGE) in regards to daily calory and fiber consumption. A significant part of the children did not reach recommendations of the consumption of macronutrients (see Table 2). The same was the case for micronutrients when analyzing only those ingested from food, but with less certainty, since information on supplements and medication was not collected (see Table 3). DGE does not specify limits for sugar consumption, but over 75% of the children consumed sugars well above the quantities recommended by the National Health Service (NHS) (24 g) [36] and the World Health Organization (WHO) recommendation of < 10% of the total energy intake to come from free sugars (appr. 30 g) [37].

**Table 2:** Daily macronutrients from food and drink as calculated from the data supplied in the 3-day food diary and compared to the recommended daily allowances (RDA) of the German Society for Nutrition (DGE). Values not meeting recommendations are highlighted in bold

*n* = 142	Min	Percentile			Max	RDA
		25	50	75		
Kilocalories	**828**	**1170**	**1308**	**1494**	2359	1500–2100
Carbohydrates %	**35.6**	**48.0**	52.2	55.8	64.1	> 50
Sugar in g	17.8	**45.3**	**56.6**	**70.8**	**157.9**	< 24 g / 10% of calories
Protein in g	**22.5 (8.0%)**	35.9 (11.9%)	42.3 (13.1%)	49.6 (14.1%)	66.5 (20.2%)	> 26 g
Fat %	**20.2**	**28.8**	32.3	**37**	**48**	30–35
multiple unsaturated fatty acids in g	0.7	2.3	3.3	4.5	8.7	
Dietary fiber in g per 1000 kcal	**1.1**	**7.8**	**9.9**	**12.1**	21.5	> 14.6

**Table 3:** Daily micronutrients from food and drink as calculated from the data supplied in the 3-day food diary And compared to the recommended daily allowances (RDA) of the German Society for Nutrition (DGE). Values not meeting recommendations are highlighted in bold. These value only show micronutrients from food, since no information on vitamin and supplement intake was collected

*n* = 142	Min	Max	Percentile			RDA
			25	50	75	
Iron (mg)	**1.0**	**2.6**	**3.2**	**4.4**	**8.0**	10
Iodine (µg)	**5.5**	**12.7**	**16.7**	**23.6**	**101.2**	140
Folate (Total Folic Acid) (µg)	**12.6**	**63.1**	**82.1**	**107.1**	220.9	180
Retinol Equivalent (ug)	**18.1**	**209.2**	471.5	1006.1	2338.6	450
Vitamin A Retinol (mg)	0.0	0.1	0.1	0.2	1.2	
Vitamin B1 (mg)	**0.1**	**0.3**	**0.4**	**0.6**	2.0	0.8–0.9
Vitamin B12 (µg)	**0.0**	**0.6**	**0.9**	**1.3**	4.3	2.5
Vitamin B2 (mg)	**0.1**	**0.2**	**0.3**	**0.4**	0.9	0.9-1.0
Vitamin B6 (mg)	**0.1**	**0.4**	**0.5**	**0.7**	1.7	1
Vitamin C (mg)	**0.6**	**30.7**	48.0	75.5	217.9	45
Vitamin D (µg)	**0.0**	**0.2**	**0.5**	**0.9**	2.7	1–2
Vitamin E Active (mg)	**0.5**	2.7	3.8	5.3	14.3	0.9-1.0
Vitamin K (µg)	**3.9**	**19.9**	**26.0**	47.4	275.0	30

The values obtained in the PAQ-C ranged between 1.56 and 4.22. The mean total PAQ-C score was 3.04 points (SD = 0.55). According to the total scores 138 (71.5%) could be classified as active and 55 (28.5%) as inactive. This data only weakly correlated with the questionnaire completed by the parents, which was returned by 167 (65%) households. In median, children were active for at least 60 min on 5 days a week (IQR: 3–7) with a median 96 min (mean 106 min) of physical activity per day (IQR: 80–122) and with 97% of children reaching the WHO recommendation of average physical activity of 60 min per day or more and thus 89% could be classified as active and 11% as inactive based on the WHO criterion of at least 60 min of physical activity every day [32]. Median daily screen time was 45 min (IQR: 30–60) and time reading or listening to stories was 30 min in median (IQR: 30–60). Children slept in median 10 h per day (IQR: 9.5–10.5). On average physical activity and sedentary behavior added up to 7.4% and 6.6% of the day, sleep accounted for 40.9% of the day and 45.4% was allocated to other activities.

Regarding the effect of COVID-19 measures on children’s sports activities, 75% of the parents reported that their children participated less or much less frequently in sports; 16.3% observed no change in the frequency of sports activities, and 8.7% reported more frequent participation. 55.8% of parents indicated that their children spent more or much more time sitting during the pandemic. 32.6% saw no change in their children’s sitting behavior and 11.6% reported that their children spent less or much less time sitting. The majority of parents (78.8%) reported no change in their children’s weight status estimate, 15.2% observed an increase (slight or significant), while a 3.9% reported their children becoming slimmer. A majority of parents (53.1%) felt the lockdown measures had no impact on the overall development of their children during the lockdown. However, 35.2% felt the measures had a negative or significantly negative impact, while only 4% reported a positive or significantly positive impact.

37.4% of housholds fell within the 4001–6000€ net income range, followed by 27.4% above 6000€, 14.0% within the 3001–4000€ range, 7.8% within the 1801–3000€ range, 3.9% below 1800€, and 9.5% chose not to disclose their income. It was possible to calculate an SES score for 159 children. The score ranged from 5 to 17 with a mean score of 14.2 (SD of 2.7) and a median of 15 (IQR: 13–16). Determinants of zBMI and zBMI change from U9 are shown in Tables 4 and 5. Nutrition, SES, parent’s anthropometrics as well as sleep and sedentary time correlated significantly with zBMI and zBMI change between U9 and the study. Explorative stepwise regression analyses for zBMI change, which analyzed variables significantly correlating with it (see Table 5) could explain 26.1% of the variance in BMI change (adjusted R² = 0.244). The model included the percentage of fat in the diet (β = 0.337, *p* < 0.001), zBMI from U9 examination (β = -0.264, *p* < 0.001), and total sleep time (β = -0.233, *p* = 0.003) as predictors. The percentage of fat in the diet has a positive association with BMI change, while baseline standard BMI and total sleep time are negatively associated. Explorative stepwise regression analyses for zBMI collected in the study, which analyzed variables significantly correlating with it (see Table 4) explains 15.0% of the variance in the dependent variable (adjusted R² = 0.132). It included three predictors: portions of cow’s milk, total drinking per day, and mother’s BMI. All predictors were significant, with cow’s milk (β = 0.240, *p* = 0.003) and mother’s BMI (β = 0.201, *p* = 0.012) as well as total drinking per day (β = 0.205, *p* = 0.011) showing a positive association with zBMI. The overall model was statistically significant (F(3, 136) = 8.023, *p* < 0.001).

**Table 4:** Correlations between zBMI and other collected variables, all listed correlations are significant (*p* < 0.05). Positive correlations indicate associations with a higher zBMI, negative correlations indicate an association with a lower zBMI. For normally distributed variables Pearsons’s correlations were calculated, Spearman correlations were calculated for non-normally distributed variables and are marked with a *

Correlations with zBMI	*R*
Anthropometrics	
Arm circumference	0.86
Abdomen circumference	0.80
Hip circumference	0.78
**Nutrition**	
**Foods**	
Cow’s milk *	0.18
Total drinking per day *	0.16
**Micronutrients**	
Saturated fatty acids *	0.17
**Socioeconomic Status**	
Socioeconomic status-rank *	- 0.18
Both parents live in the same household *	- 0.19
**Other**	
BMI of father	0.28
BMI of mother	0.24

**Table 5:** Correlations between zBMI change and other collected variables, all listed correlations are significant (*p* < 0.05). Positive correlations indicate associations with a higher zBMI, negative correlations indicate an association with a lower zBMI. For normally distributed variables Pearsons’s correlations were calculated, Spearman correlations were calculated for non-normally distributed variables and are marked with a *

Correlations with zBMI change U9 to study	*R*
Anthropometrics	
zBMI	0.35
zBMI U9	-0.37
**Nutrition**	
**Macronutrients**	
Fat percentage	0.36
Carbohydrates percentage	-0.36
Fat (g)	0.28
**Micronutrients**	
Saturated fatty acids percentage	0.20
Vitamin A Retinol (mg) *	-0.21
Mono-unsaturated fatty acids	0.17
**Physical Activity**	
Screen time *	0.26
Total sedentary time *	0.22
Total sleep time *	-0.21
**Other**	
Height mother *	-0.17
Age father *	0.18

## Discussion

This study aimed to assess the prevalence of overweight and obesity, physical activity and eating habits as well as the retrospective anthropometric development in the context of COVID-19 containment measures among a sample of primary school children (second grade) in the Rhein-Neckar Region, Germany. The prevalence of obesity in this group was 5%, 5% for overweight, 75% for normal weight and 16% for underweight. The mean zBMI of -0.25 that was significantly lower than the reference population and a subgroup with national U-Examinations available [34] showed a significant zBMI decrease between last examination (U9) and the study, which was performed towards the end of COVID-19 pandemic measures. While 89% of the children, for whom physical activity questionnaires were returned (*n* = 165) fulfilled WHO recommendations for physical activity, a majority of children for whom food diaries were available (*n* = 142) did not reach age-adjusted nutritional recommendations of the German Society for Nutrition (DGE) in regards to total calories, macronutrients and possibly micronutrients. These results contradict parents’ perceptions, who reported their children to have disproportionally gained weight, done less sports and engaged more often in sedentary behavior.

The KiGGS study, performed between 2014 and 2017 estimated that 9,5% of the children in Germany were overweight and 5.9% obese [3] and school enrollment examinations from 2006 in the Rhein-Neckar-Region showed a slightly lower prevalence with 6.0% for overweight and 4.2% for obesity [38], similar to the 5.1% overweight and 4.7% obesity in this study. Contrary to some previous publications [19, 39–41] and the perception of parents of participating children, the study participants appeared to have lost weight in the time which involved the lockdown measures. The decrease in weight cannot be considered healthy, since there was a significant zBMI decrease in normal weight children, despite living in households with above average net incomes and socioeconomic status (65% of study participants reported an income of > 4.000€, mean German household income 3.800€ [42]).

According to the explorative regression analyses for zBMI at the timepoint of the study milk consumption, overall drinking quantity and BMI of the mother were identified as significant predictors (all three relationships positive). The regression analysis for zBMI change showed that a low percentage of fat in the diet, longer sleep and lower zBMI in the baseline examination (U9) was associated with a decrease in zBMI.

Since zBMI did not significantly differ between U8 and U9 examinations yet decreased significantly from the U9 examination to the study, pandemic measures in between may have contributed to this change. It is also worth noting that in this cohort, underweight was more prevalent than overweight and obesity combined (16% vs. 10%). The data collected does not provide explicit answers as to why this may have been the case, however the low calory consumption and low quality of the diets as well as a relatively high percentage of physically active children likely contributed to the changes observed. Subjective questions with respect to the effect of the pandemic on weight development and physical activity of the children hint at parents anticipating or noticing a decreased physical activity in their children, and possibly adjusting the diets of the children as to prevent an anticipated weight gain (more parents had an impression that their child gained weight than otherwise).

It is important to note the study results cannot be generalized to all children, since the majority of children participating in this study came from families with a relatively high SES. The voluntary participation without any incentive for parents likely contributed to an underrepresentation of children with lower economic status or a migration background. As a consequence of low participation, the study did not reach its recruitment goal of 369 participants based on sample size necessary to determine the obesity prevalence with sufficient power. In addition, it is unclear how standardized the pediatricians conducted the U8 and U9 examinations, which poses a further limitation of the longitudinal analysis of zBMI in this study. Questionnaires were designed or adapted for this study, since using only validated or previously published questionnaires would have further increased the quantity of questions, parents had to complete and was considered a hurdle for study participation. It was clear that the PAQc is not the optimal tool in this age group, yet it was applied due to a lack of suitable alternatives. The data suggests, it was not useful at all, since there were almost no associations with parent reported physical activity found.

It has been shown that children in western populations regularly do not achieve goals set by dietary guidelines [43]. And children from a high socioeconomic background do not appear to have issues with excess body weight. There are many other potential explanations, which may have contributed to the zBMI development in this study independent of the pandemic. For example, children going to kindergarten in Germany are provided with food, but not all children in Baden-Württemberg going to primary school eat regular lunch, since food is usually subsidized but not provided by the government, as is the case in many other countries. U8 and U9 examinations take place when children are still in kindergarten while this study was performed during the 2nd year at school, which could be an explanation for the decrease in zBMI observed. Insufficient food intake is associated with poorer cognitive functioning, decreased school attendance, lower achievement as well as iodine and possibly iron deficiency [44]. Dietary quality and diversity appear to be predictors for academic performance [45, 46] and BMI development [47–49]. The results from this study show that there is room for improvement of the dietary quality of children in Germany even in households with a high SES. Based on previous data it can only be anticipated that this will also be the case in children from a lower socioeconomic background. Improving diet quality could for example be done by implementing regular and healthy school meals in German primary schools. Regular school meals have been shown to consequently improve cognitive function, school performance, BMI [50–54] and quality of life [55, 56].

## Conclusions

Contrary to observations in other populations and parent perception, primary school children with a high socioeconomic background in Germany appear to have significantly lost and not gained weight during the COVID-19 pandemic with a significant increase in the proportion of underweight children. This observation appears to be mainly driven by inadequate nutrition.

### Electronic supplementary material

Below is the link to the electronic supplementary material.

Supplementary Material 1

## Data Availability

The datasets during and/or analyzed during the current study available from the corresponding author on reasonable request for scientific purposes.

## Abbreviations

BMI: Body Mass Index
DGE: Deutsche Gesellschaft für Ernährung (German Society for Nutrition)
IQR: Interquartile range
NHS: National Health Service (of the United Kingdom)
PAQ-C: Physical Activity Questionnaire for Children
SES: Socioeconomic status
WHO: World Health Organization
U8: Standardized medical exam for appr. 4-year-old children in Germany
U9: Standardized medical exam for appr. 5-year-old children in Germany
zBMI: Age and sex standardized z-score of Body Mass Index
