# Supplementary material for: Impact of Covid-19 lockdowns on the anthropometric development in primary school children in the Rhein-Neckar Region, Germany
Source: BMC Nutr. 2024 May 29;10:78. doi: 10.1186/s40795-024-00886-2 (PMC11134761; doi:10.1186/s40795-024-00886-2)
Supplement: Supplementary file 1 — Supplementary Material 1 [file 40795_2024_886_MOESM1_ESM.pdf]

6. Fragebogen sozioökonomischer Status und kultureller Hintergrund (Dauer ca. 3 Minuten)

Diesen Fragebogen benötigen wir, da auch der soziale und ökonomische Hintergrund bei unserer Fragestellung eine wichtige Rolle spielt. Wenn Sie nicht bereit sein sollten, diesen Fragebogen auszufüllen, vermerken Sie dies bitte kurz formlos auf dieser Seite, so dass wir von Rückfragen zu diesem Fragebogen absehen.

Bildung und berufliche Qualifikation der Eltern:

1. Höchster Bildungsabschluss der Mutter (bitte ankreuzen):

- o Kein Schulabschluss
- o Hauptschule (oder vergleichbar)
- o Mittlere Reife (oder vergleichbar)
- o Lehre/Berufsschule (oder vergleichbar)
- o allgemeine/fachgebundene Hochschulreife/Abitur (oder vergleichbar)
- o Hochschule / Universität (oder vergleichbar)
- o Keine der oben genannten Optionen trifft zu

2. Höchster Schulabschluss des Vaters (bitte ankreuzen):

- o Kein Schulabschluss
- o Hauptschule (oder vergleichbar)
- o Mittlere Reife (oder vergleichbar)
- o Lehre/Berufsschule (oder vergleichbar)
- o allgemeine/fachgebundene Hochschulreife/Abitur (oder vergleichbar)
- o Hochschule / Universität (oder vergleichbar)
- o Keine der oben genannten Optionen trifft zu

3. Aktuelles Nettoeinkommen des Haushaltes in €/ Monat (geschätzt)

- o <1800 €
- o 1801 - 3000 €
- o 3001 € - 4000 €
- o 4001 € - 6000 €
- o > 6000 €
- o Keine Angabe

4. Leben beide Eltern mit dem Kind in einem Haushalt?

- o Ja
- o Nein

5. Geburtsland Kind

- o Deutschland
- o Anderes Land, welches?______________________________

6. Wenn Ihr Kind nicht in Deutschland geboren ist, seit wie vielen Jahren lebt Ihr Kind in Deutschland?

____

7. Geburtsland Vater

- o Deutschland
- o Anderes Land, welches? __________________________________

8. Geburtsland Mutter

- o Deutschland
- o Anderes Land, welches? __________________________________

9. Wird zuhause Deutsch gesprochen?

- o Ausschließlich Deutsch
- o Größtenteils Deutsch
- o Ungefähr zur Hälfte Deutsch
- o Größtenteils eine andere Sprache
- o Ausschließlich eine andere Sprache

«Pseudonym»

6. Socioeconomic status and cultural background questionnaire (appr. 3 min.)

Previous studies show that socioeconomic status as well as cultural background have an influence on the weight status and weight development. Should you not be willing to complete this questionnaire, please make a quick note of it here and send it back to us with the other documents. This way, we will know the data is not available and will refrain from making further inquiries.

Education and professional qualification of the parents::

1. **Highest educational level / qualification of mother (please tick one):**

- o No school degrees
- o Secondary modern school or similar (equivalent to appr. 8 classes)
- o Secondary school or similar (equivalent to appr. 10 classes)
- o Professional education or similar (e. g. finished apprenticeship)
- o General qualification for university entrance or similar (e. g. A-levels, Abitur etc.)
- o University degree, college degree or similar
- o Other (above options are not applicable)

2. **Highest educational level / qualification of the father (please tick one):**
3. No school degrees
4. Secondary modern school or similar (equivalent to appr. 8 classes)
5. Secondary school or similar (equivalent to appr. 10 classes)
6. Professional education or similar (e. g. finished apprenticeship)
7. General qualification for university entrance or similar (e. g. A-levels, Abitur etc.)
8. University degree, college degree or similar
9. Other (above options are not applicable)

10. **Current net income of the household per month, estimated in €**

- o <1800 €
- o 1801 - 3000 €
- o 3001 € - 4000 €
- o 4001 € - 6000 €
- o > 6000 €
- o Not available

«Pseudonym»

11. Do both parents live in the same household?

- Yes
- No

12. Child's country of birth

- Germany
- Other country, which?______________________________

13. If your child was not born in Germany, since how many years does he/she live in Germany?

____

14. Country of birth father

- Germany
- Other country, which?______________________________

15. Country of birth mother

- Germany
- Other country, which?______________________________

16. Is German spoken at home?

- German only
- German mostly
- German half of the time
- Mostly another language
- Only another language
